# Design and Optimization of a Hybrid Design for Quantum Transduction

**DOI:** 10.3390/s25206365

**Published:** 2025-10-15

**Authors:** Enrico Bargagna, Julian Delgado, Changqing Wang, Ivan Gonin, Vyacheslav P. Yakovlev, Paolo Neri, Donato Passarelli, Silvia Zorzetti

**Affiliations:** 1 DICI—Department of Civil and Industrial Engineering, University of Pisa, Largo Lucio Lazzarino 1, 56122 Pisa, Italy; e.bargagna2@studenti.unipi.it; 2 Fermi National Accelerator Laboratory, Batavia, IL 60510, USA; jdelgado@fnal.gov (J.D.); cqwang@fnal.gov (C.W.); gonin@fnal.gov (I.G.); yakovlev@fnal.gov (V.P.Y.); donato@fnal.gov (D.P.); zorzetti@fnal.gov (S.Z.)

**Keywords:** quantum transduction, quantum computing, sensing

## Abstract

This study presents the mechanical design and analysis of a quantum electro-optical transducer engineered to operate at millikelvin temperatures within a dilution refrigerator. The transducer enables bidirectional microwave-optical frequency conversion through a hybrid architecture that integrates a superconducting radiofrequency (SRF) cavity with an electro-optic optical cavity. Among several design options investigated, the configuration offering the best thermal and mechanical performance was selected, yielding a robust solution with reduced sensitivity to fabrication tolerances, improved heat dissipation, as well as alignment precision. The design ensures uniform temperature distribution, enabling higher laser pump powers and, thus, increased conversion efficiency, while maintaining mechanical stresses safely below the material yield strength. Electromagnetic simulations further validate the design, demonstrating enhanced coupling between the optical and microwave modes, as well as a broader tuning range achieved with smaller tuner displacements.

## 1. Introduction

Among the various platforms for quantum computing, superconducting qubits stand out for their scalability and ability to perform high-fidelity quantum operations [1,2]. However, their operation is restricted to the microwave domain, posing significant challenges for large-scale quantum networks. Microwave photons are subject to detrimental thermal noise, making long-distance quantum communication impractical [3]. Optical photons, with frequencies in the hundreds of terahertz (THz), possess higher energies and are immune to thermal noise at room temperature. Additionally, they can be efficiently transmitted through optical fibers outside cryogenic environments, making them a promising solution for quantum communication and the interconnection of distant quantum nodes. Microwave-optical quantum transducers play a key role in bridging the energy gap between microwave and optical photons, which spans approximately five orders of magnitude [4,5,6], thus, enabling the seamless integration of optical communication systems with superconducting quantum devices. The design analyzed in this study is based on an electro-optic optical cavity coupled to a superconducting radiofrequency (SRF) cavity, with a geometry analogous to that in Ref. [7], where considerations of the device are reported regarding the characterization of quantum mechanical properties such as coherence time and the quantum entanglement. Low-energy microwave photons are converted into high-energy optical photons. An optical pump mediates the process. The optical pump, provided by the laser source, supplies both the energy required for conversion and a reference frequency. Typically, the efficiency of the up-conversion process can be increased by increasing the pump power. However, part of the optical pump energy is dissipated in the cavity, which can lead to device heating, conflicting with cryogenic requirements for host superconducting qubits [8]. A transducer is a hybrid device, integrating different materials which are not well-characterized in the quantum regime—i.e., at single photon level and millikelvin temperatures, as concerns superconducting qubits. The introduction of mechanically compliant elements in this work reduces the impact of component tolerances, enhancing mechanical stability and experimental reproducibility. These advancements contribute to the development of high-efficiency quantum transduction, facilitating the integration of superconducting qubits into large-scale quantum networks.

This work focuses on enhancing transducer performance by improving the dissipation of the heat generated by the optical pump. Furthermore, the proposed solution ensures crystal alignment within the SRF cavity. These are two key factors that enable operations at lower temperatures and with strong electro-optic coupling.

## 2. Materials and Methods

### 2.1. Overview of the Transducer Design

The quantum transducer analyzed in this study is a hybrid device based on a three-dimensional architecture. The basic design consists of a bulk SRF cavity integrated with a lithium niobate optical resonator [7].

**Figure 1:**
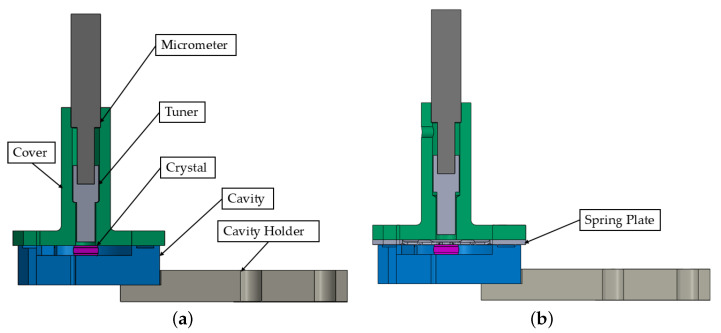
Finite Element (FE) model sectional views of the transducer design and main components. (**a**) Main components of the transducers. (**b**) A spring plate is added to the design.

Key components of the transducer are highlighted in Figure 1a. The system is designed to operate at millikelvin temperatures within a dilution refrigerator, with laser pumping applied to excite the optical mode on the rim of the polished lithium niobate crystal. The crystal is enclosed by an SRF cavity, which is fabricated from niobium or aluminum.

The resonant frequency of the microwave dipole mode can be tuned by adjusting the vertical position of a tuning mass.

The cavity holder is made of oxygen-free high thermal conductivity (OFHC) copper (UNS C10100) and serves as the mounting interface between the RF cavity and the dilution refrigerator’s mixing chamber (MXC) cold plate. The tuner is made from a Copper mass press-fit onto a single-crystal sapphire (Al_2_O_3_) rod to minimize the electromagnetic losses in the RF cavity. Further details on material properties at cryogenic temperatures are provided in Appendix A.

### 2.2. Simulation Setup and Boundary Conditions

Electromagnetic, static structural, and thermal analyses were performed in Ansys Workbench to investigate the temperature distribution and mechanical stresses under optimal operating conditions. Through these multi-physics analyses, the impact on the microwave mode is also evaluated.

A tolerance analysis was conducted to assess the impact of dimensional variations on the system’s temperature distributions and mechanical stresses. We focus on the contact between the crystal and the cover, which is particularly relevant for this hybrid device.

The preliminary evaluation highlighted the sensitivity of the temperature and stress distribution to component tolerances, as well as the importance of ensuring proper crystal centering within the SRF cavity. To enhance these aspects, a new component spring plate has been introduced between the cover and the cavity, as shown in Figure 1b.

**Figure 2:**
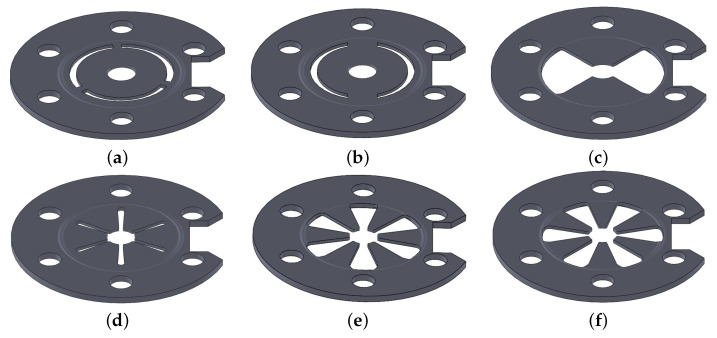
Proposed designs of the spring plate: (**a**) three circumferential slots; (**b**) two circumferential slots; (**c**) two radial holes; (**d**) six radial holes; (**e**) six radial enlarged holes; (**f**) six radial enlarged holes rotated by 30°.

Several geometries for the design of the spring plate were analyzed, as shown in Figure 2. The design goal of these versions is to ensure contact between the crystal and the cover in any configuration, despite the unavoidable dimensional tolerances of the components. The contact must remain sufficiently compliant to prevent excessive stress concentrations in the crystal caused by contact forces. Thus, the idea was to introduce a compliant plate with moderate stiffness to avoid stress-related issues. The cuts present in the proposed designs serve to reduce the bending stiffness of the plate.

**Figure 3:**
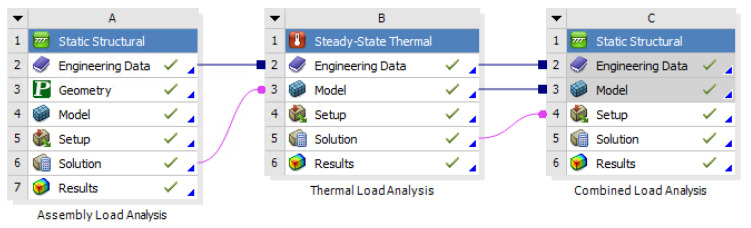
Schematic of the developed FE model.

To ensure contact between the crystal and the spring plate, the recess of the cavity supporting the cover component is reduced by at least the height tolerance of the crystal. This adjustment allows the spring plate to deform during bolt tightening in the assembly process at room temperature, such that it is in contact with the crystal before fully engaging with the contact surface of the cavity component. Thermal and mechanical analyses were conducted to evaluate the effects of thermal deformations on the stress state of the crystal. A schematic view of the model is shown in Figure 3.

The assembly process was simulated by applying a downward displacement to the cover of the cavity to ensure contact with the cavity. The spring plate is already in contact with the crystal in the first simulation step. The simulations focused on the most critical scenario, in which the measured height of the crystal is at its maximum value within the fabrication tolerance range, while the cavity depth offset is at its minimum. The laser pump is modeled as a heat source occupying the same volume as the optical mode in the crystal, approximately 10 μm around the equator [8]. An initial temperature of 7 mK was assumed for the thermal simulations. Two boundary conditions were applied: (i) internal heat generation within the crystal and (ii) a constant temperature at the surface of the cavity holder, considered in contact with the dilution refrigerator.

Additionally, a radio-frequency electromagnetic analysis was conducted to evaluate the resonance frequency of the transducer system and the frequency tuning range provided by the tuner. The model consists of three main components: the crystal, the waveguide chip that couples the pump to the crystal, and the vacuum space confined within the transducer surfaces.

The electromagnetic resonance frequency is determined by the capacitance and the inductance of the effective cavity circuit, which effectively depend on: the geometry of the cavity, the position of the antennas, and the dielectric properties of the crystal and waveguide chip.

The distance between the tuner and the crystal is varied to tune the resonant frequency; in this design, it spans a range up to 5 mm. The final mesh was obtained through a convergence analysis, ensuring that the simulation results were independent of the mesh size. Two boundary conditions were applied to simulate the behavior of the antennas. These conditions consisted of imposing zero resistance and a reactance equal to the impedance of free space. For the evaluation of the resonance frequency, only the dipole mode with the axis aligned with the symmetry plane of the cavity was considered. The reason for focusing exclusively on the dipole mode lies in the operation of the electro-optic transduction process. Information is transferred from the microwave mode to the infrared optical mode, which acts as a whispering gallery mode where the light propagates along the surface of the crystal. The microwave mode, on the other hand, oscillates both in the cavity and within the crystal. In the three-way mixing process, two initial fields—one infrared and one radio-frequency—combine to generate a third infrared field that is modulated by the microwave frequency.

### 2.3. Design Considerations and Rationale

To optimize the transducer’s performance, the mechanical bending stiffness of six different spring plate designs was evaluated. The primary objective of this analysis was to minimize the stresses on the crystal to prevent potential damage that could compromise both its structural integrity and optical quality factor.

The equivalent stiffness of each design was first calculated with a static structural analysis, and the results are summarized in Table 1. The comparison reveals that designs “e” and “f” yield lower equivalent stiffness, which results in reduced stresses on the crystal.

**Figure 4:**
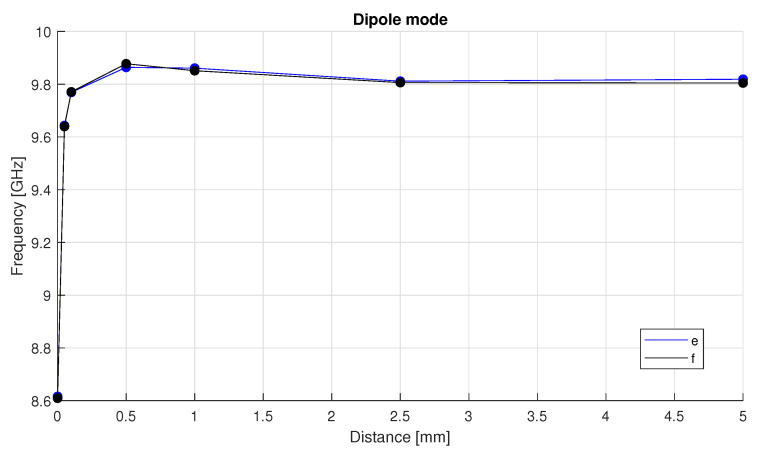
Trend of the resonance frequency of the dipole mode of the proposed designs as a function of the tuner’s distance from the crystal.

To further refine the selection, electromagnetic field simulations were performed for both versions, “e” and “f”. The results, presented in Figure 4, show that both versions exhibit a similar trend in resonance frequency as a function of the tuner’s distance from the crystal.

Beyond the resonance frequency response, the performance of the two designs was further evaluated by calculating the single-photon electro-optic coupling strength $g_{\mathrm{eo}}$ , defined as:

(1) $$g_{\mathrm{eo}}=\frac{1}{16\pi }n^{2}r_{33}\sqrt{\omega_{p}\omega_{a}}\sqrt{\frac{\hbar \omega_{b}}{W}}\int_{0}^{2\pi }\frac{E_{\mathrm{RF}}\left(\phi \right)}{\cos\theta }\;d\phi$$

**Figure 5:**
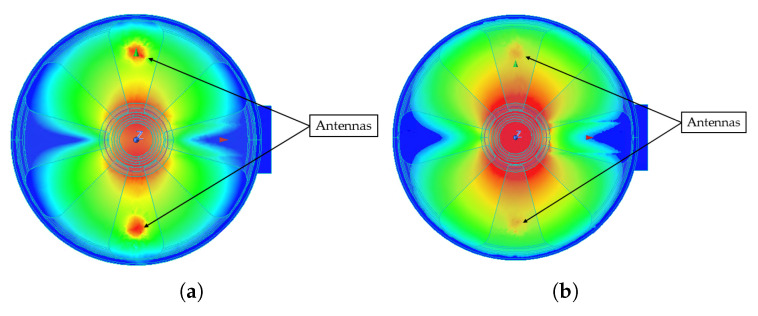
Electric field distribution of the dipole mode within the cavity volume: (**a**) refers to the “e” version, while (**b**) corresponds to the “f” version.

Here, *n* is the extraordinary refractive index of lithium niobate near 1550 nm, and $r_{33}$ is the linear electro-optic coefficient of lithium niobate. The pump frequency is given by $\omega_{p}=\frac{c}{1550\times 10^{-9}}$ , while $\omega_{b}$ is the microwave frequency, and $\omega_{a}=\omega_{p}+\omega_{b}$ denotes the sideband frequency of the converted optical photon. The parameter *W* represents the total microwave energy stored in the RF cavity. The term $\frac{E_{\mathrm{RF}}}{\cos\theta }$ describes the electric field normalized to the number of microwave photons, determined by the stored energy (*W*). Comparing the integral $\int_{0}^{2\pi }\frac{E_{\mathrm{RF}}\left(\phi \right)}{\cos\theta }\;d\phi$ , the “f” configuration results in a slightly higher value of $4.560\times 10^{9}$ , than the $4.559\times 10^{9}$ “e” configuration. This slight difference is attributed to the distribution of the electromagnetic field intensity, as shown in Figure 5. In the “e” design, the electric field tends to concentrate near the antenna regions, leading to a reduction in the field strength within the crystal and, consequently, a lower $g_{\mathrm{eo}}$ . In contrast, in the “f” configuration, the field is more uniformly distributed, with stronger fields within the crystal, thereby enhancing the coupling strength.

After identifying the “f” design as the most suitable option, additional grooves were introduced to further reduce its stiffness. These modifications resulted in an equivalent bending stiffness of 192 N/mm for the component, effectively minimizing the contact forces exerted on the crystal.

**Figure 6:**
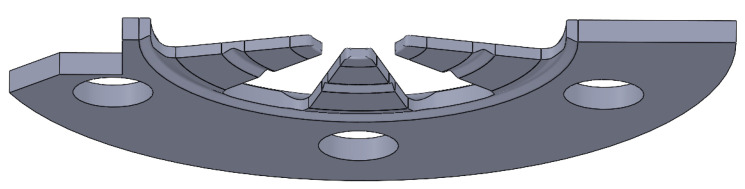
Sectional view of the spring plate, highlighting its internal structure and key geometric features.

A sectional view of the component is provided in Figure 6 to highlight its geometry and the implemented design optimizations.

## 3. Results

This section presents the results of thermal, mechanical, and electromagnetic simulations for both the preliminary design and the proposed enhanced design with the spring plate. Each analysis aims to assess the performance improvements achieved through the design modifications. The results are complemented by graphical representations of temperature distribution, mechanical stress, and electromagnetic field, as well as comparative tables summarizing key parameters for a laser input power of 1 mW.

### 3.1. Thermal Analysis

The thermal behavior was assessed by analyzing the temperature distribution on the crystal, cover, and cavity. Simulations were conducted by applying a constant temperature of 7 mK to the surface of the cavity holder, which is thermally anchored to the plate of the dilution refrigerator, and an input laser power of 1 mW at the equatorial plane of the crystal.

**Figure 7:**
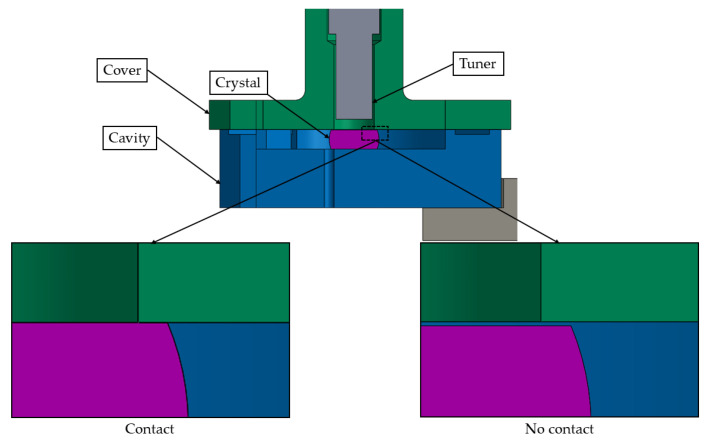
Sectional view of the model along with detailed views of the components, highlighting the two configurations: with and without contact.

Initially, a tolerance analysis was performed to evaluate the impact of dimensional variations in the crystal on the temperature distribution for the preliminary design, excluding the spring plate. Two configurations were considered: one with contact and one without contact between the crystal and the cover. These configurations correspond to the maximum and minimum crystal height within the fabrication tolerance range, as shown in Figure 7. In the non-contact condition, the gap between the crystal and the cover is approximately 40 µm.

**Figure 8:**
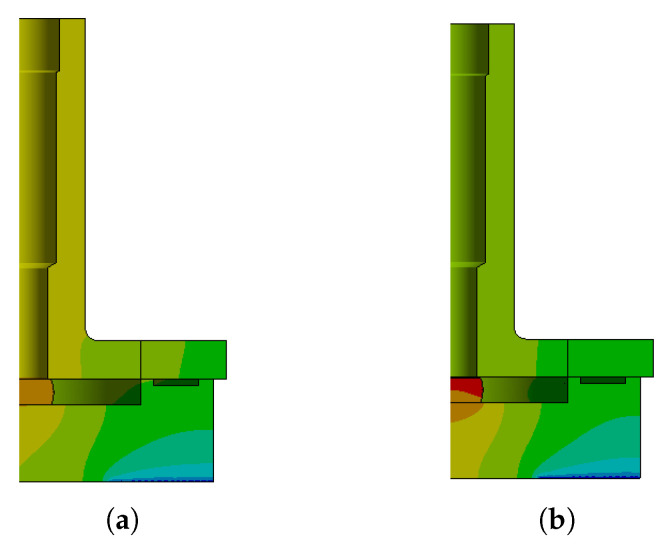
Temperature distribution of two configurations without the spring plate: (**a**) contact; (**b**) non-contact.

The temperature distribution for both configurations is shown in Figure 8, with the configuration featuring contact on the left and the configuration without contact on the right. It is observed that ensuring contact between the crystal and the cover results in lower and more uniform temperatures across the device. In contrast, the configuration without contact leads to significant thermal gradients, particularly within the crystal. With an input power of 1 mW, the average crystal temperature increases by approximately 50 mK when comparing the configurations with and without contact.

**Figure 9:**
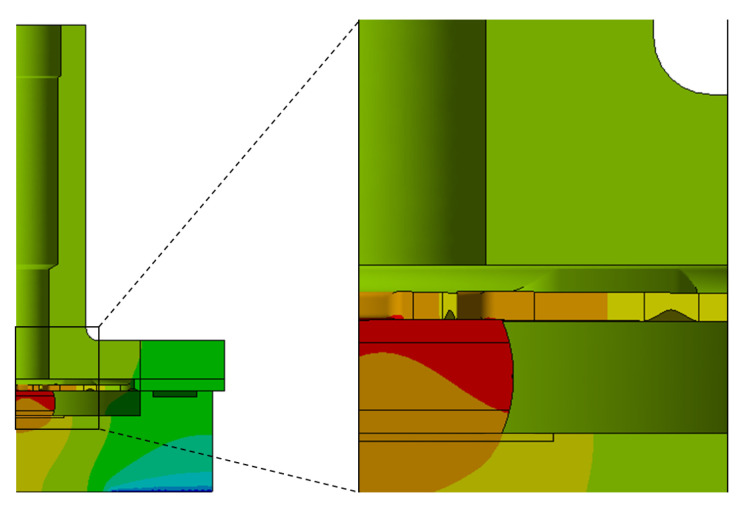
Temperature distribution of the proposed design with 1 mW laser power.

We also evaluated the temperature distribution for the design with the spring plate (Figure 9). It is noted that this design always ensures contact with the top of the crystal, making the temperature distribution less sensitive to the precise dimensions of the components. The mean temperatures are normalized with respect to the mean cavity temperature for both configurations; the results are provided in Table 2. For the design without the spring plate, the temperature values correspond to the worst-case scenario, where the crystal is not in contact with the cover. The design with the spring plate exhibits lower mean temperatures for both the crystal and the cavity, highlighting its higher thermal management capabilities.

### 3.2. Mechanical Analysis

**Figure 10:**
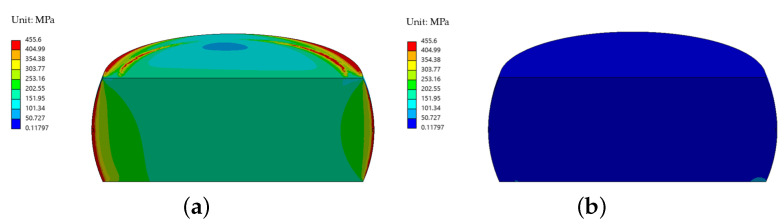
Stress distribution of the preliminary design with a 1 mW laser power: (**a**) case with contact and (**b**) case without contact.

Mechanical simulations were performed to evaluate the Von-Mises stress distribution within the device under thermal loading. The simulations indicate that the stress distribution in the components is weakly dependent on the laser power within the range of 1 µW to 2 mW. The stress distribution for the preliminary design is shown in Figure 10, considering both cases: with contact between the crystal and the cover, as described in the thermal analysis of the previous section (Figure 10a), and without contact (Figure 10b).

The configuration in which the crystal is in contact with the lid represents the most critical condition in terms of mechanical stress, whereas negligible stress levels are observed when there is no contact, as expected since the crystal does not bear any mechanical load in this case. In this scenario, the equivalent maximum stress in the crystal reaches 455.6 MPa.

In the proposed design with the spring plate, the stiffness of the system is intentionally reduced to accommodate the dimensional variations of the crystal within the specified tolerances, while maintaining structural integrity. This approach effectively mitigates the stress induced by the force fit.

**Figure 11:**
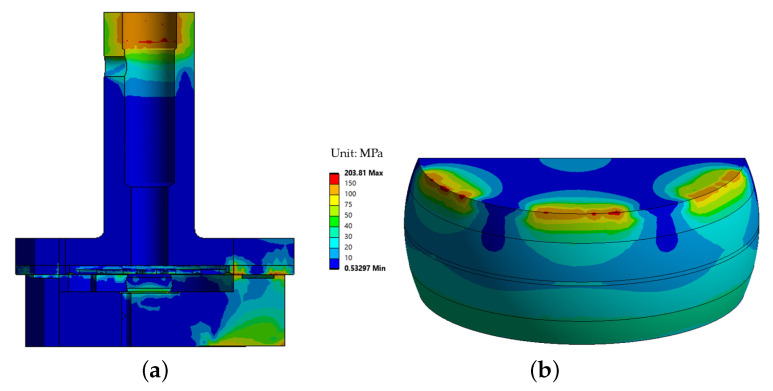
Stress distribution in the enhanced design: (**a**) overall view of the device and (**b**) detailed view of the crystal.

The introduction of a compliant plate mitigates these stresses by reducing contact rigidity and stress levels in the crystal. The stress distribution in the enhanced design is shown in Figure 11, which includes (a) a full view of the device and (b) a detailed view of the crystal, where the equivalent maximum stress in the crystal reaches 203.81 MPa.

### 3.3. Electromagnetic Analysis

**Figure 12:**
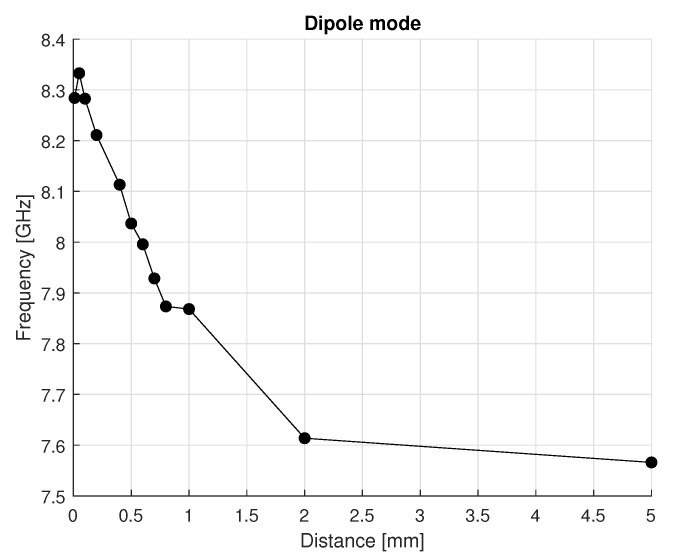
Resonance frequency of the current design of the dipole mode as a function of the tuner’s distance from the crystal.

The electromagnetic field distribution analysis was performed to evaluate the resonance frequency of the transducer and the frequency tuning range provided by the tuner for the dipole mode. The simulations indicate that the cavity’s frequency tuning range is ±0.379 GHz (moving the tuner for its whole stroke) (Figure 12). Additionally, it can be observed that the resonance frequency is highly sensitive to the tuner’s distance for values lower than 2 mm, beyond which the frequency nearly saturates and the tunability of the transducer is significantly reduced.

Similarly, electromagnetic field analysis was performed to assess the resonance frequency and tuning range of the design with the spring plate. The material properties and boundary conditions are the same as the preliminary design simulations. The simulations indicate that the frequency tuning range of ±0.694 GHz, as shown in Figure 4. Furthermore, it can be observed that the frequency is highly sensitive to the tuner’s distance for values lower than 0.5 mm, beyond which the frequency nearly saturates, and the tunability of the transducer is significantly reduced.

The electromagnetic results in Table 3 also report the evaluation of the integral of the ratio between the vertical electric-field component and the cosine of the curvilinear coordinate along the crystal perimeter confining the optical mode, as well as the conversion efficiency estimate. In the steady-state regime, the system dynamics yield identical transfer functions for bi-directional conversion between microwave and optical signals due to reciprocity. The bi-directional frequency conversion efficiency depends on the microwave–optical cooperativity and losses:

(2) $$\eta =\frac{\gamma_{a,c}}{\gamma_{a}}\frac{\gamma_{b,c}}{\gamma_{b}}\;\cdot \;\frac{4C}{{\left(1+C\right)}^{2}},\;C=\frac{4n_{p}g_{eo}^{2}}{\gamma_{a}\gamma_{b}},$$

where *C* represents the cooperativity between the optical and microwave modes, which depends on the photon number in the pump mode ( $n_{p}$ ). The second factor of Equation (2) corresponds to the internal efficiency $\eta_{i}=4C/{\left(1+C\right)}^{2}$ , which approaches unity for C=1 . For a generic *m* mode (m=a,b) , $\gamma_{m}$ is the total loss rate, consisting of both the internal loss and external (coupling) loss:

(3) $$\gamma_{m}=\gamma_{m,o}+\gamma_{m,c}$$

where $\gamma_{m,o}$ is the internal loss and $\gamma_{m,c}$ is the external (coupling) loss. A detailed description of the variables is provided in Ref. [8]. The results confirm that the structure with the compliant plate offers two key advantages: a wider tuning range and a higher conversion efficiency.

## 4. Discussion and Conclusions

In this paper, a hybrid device for quantum transduction was analyzed and optimized through mechanical, thermal, and electromagnetic simulations. The proposed improvements aim to enhance the device’s thermal stability, mechanical reliability, and conversion efficiency. The analyses confirm that the proposed design, featuring a compliant spring plate, maintains stress levels below the yield limits despite tolerance-related dimensional uncertainties, unlike the previous configuration. Furthermore, it ensures proper centering of the crystal within the cavity and stabilizes the average crystal temperature. This design also prevents any displacement of the crystal during operation or handling of the device during assembly in the refrigerator.

The introduction of the proposed spring plate provides stable contact at the top of the crystal, enhancing heat dissipation and reducing the sensitivity to geometric variations. The stress levels in the crystal remain non-negligible relative to the yield limit, which could impact long-term fatigue life. To mitigate this issue, the bending stiffness of the introduced component would be further reduced, potentially by decreasing its thickness, while carefully considering the constraints imposed by machining tolerances and manufacturing feasibility. At this stage of the research, the fatigue life of the crystal was not evaluated, as load cycles occur only during subsequent cool-downs and warm-ups of the cavity, which are infrequent (each cool-down process lasts up to 20 h). Consequently, fatigue failure was not considered a critical factor in this study.

Regarding the tuning range, a significant improvement over the preliminary design is observed, up to ±300 MHz. This improvement enables higher operating powers, which increase conversion efficiency and enhance the quality factor. A wider tuning range also provides greater flexibility, enabling the design to better adapt to incoming signal disturbances. Furthermore, the higher value of the integral of the vertical electric field component normalized by the cosine of the curvilinear coordinate—evaluated along the perimeter where the whispering gallery mode is confined—strengthens the optical–microwave coupling, thus, leading to improved conversion efficiency.

**Figure 13:**
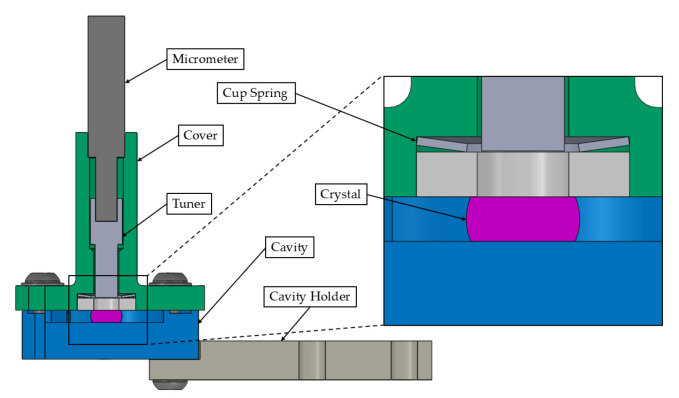
New design idea with a cup spring in the cover.

The main limitations of this study stem from approximations in the analysis, such as the extrapolation of material properties to cryogenic temperatures and the neglect of thermal contact resistances between components, which is a common approach in the literature. These factors could significantly affect the temperature distribution and the resulting mechanical stresses, so a more detailed evaluation could improve prediction accuracy and will be the subject of future research. To further reduce the operating temperature and enhance performance, a thermalization structure could be introduced. This would involve the addition of high-thermal-conductivity materials to link areas subject to higher thermal loads, such as the cavity, to cold regions like the plate of the dilution refrigerator. Potential limitations in thermal contact could be mitigated by employing copper thermal straps or various interposers and filler materials, such as thermal greases and varnishes. An alternative transducer design, shown in Figure 13, could offer similar advantages to the one proposed in this paper. This design utilizes a beryllium copper cup spring, which would act as low-stiffness elements to avoid stresses at the contact points between the crystal and other components. The choice of beryllium copper is driven by the excellent mechanical and electrical properties of this alloy. Composed of a small amount of beryllium (typically between 0.5% and 2.5%), beryllium copper is well known for its high mechanical strength, which exceeds that of pure copper, making it particularly suitable for applications requiring resistance to mechanical stress, such as springs and structural components. Despite its high mechanical strength, beryllium copper retains good electrical conductivity (although lower than pure copper), making it useful for electronic and electrical applications where both mechanical strength and electrical conductivity are required. Additionally, its non-ferromagnetic nature is beneficial in applications where avoiding magnetic interference is critical, such as in electronic devices and communication systems. While this alternative solution offers simpler modifications from a technological standpoint, it introduces new challenges. Specifically, the numerous sliding contacts created by the spring elements could complicate thermal evaluation, as the thermal contact resistances between these elements and the other components are difficult to assess. Moreover, the modification of the cover component, although less complex in terms of manufacturing processes, would still require careful consideration of these additional contacts.

## Figures and Tables

**Table 1:** Equivalent stiffness values for the six proposed spring plate designs.

Equivalent Stiffness (N/mm)	a	b	c	d	e	f
k_*equivalent*_	322.8	285.9	244.8	420.6	228.8	228.8

**Table 2:** Comparison of the normalized mean temperatures of key components, normalized with respect to the mean cavity temperature.

Designs	Crystal	Cavity	Cover & Plate
w/o spring plate	1.483	1	1.004
w/ spring plate	1.37	1.012	1.078

**Table 3:** Comparison between the preliminary and enhanced transducer design.

Designs	Tuning Range (GHz)	$\int_{0}^{2\pi }\frac{E_{\mathrm{RF}}\left(\phi \right)}{\cos\theta }\;d\phi$	η
w/o spring plate	±0.379	$3.70\times 10^{9}$	48.36%
w/ spring plate	±0.694	$4.56\times 10^{9}$	54.02%

**Figure A1:**
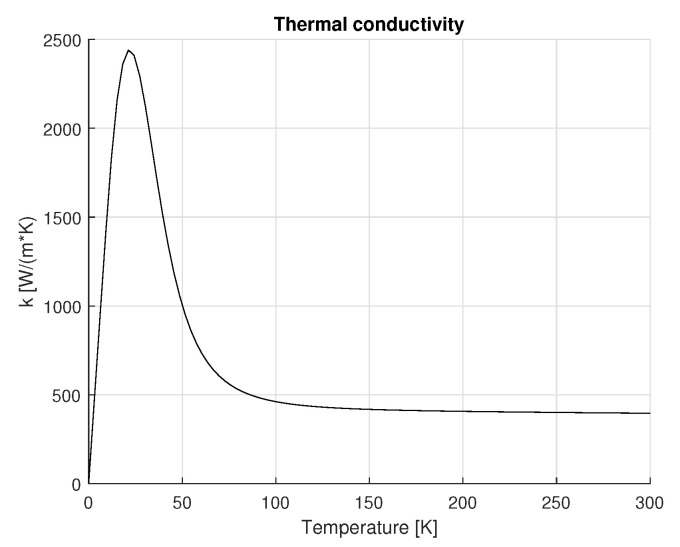
Graph of the thermal conductivity of OFHC Copper (UNS C10100)-100RRR as a function of temperature [10,11,12].

**Figure A2:**
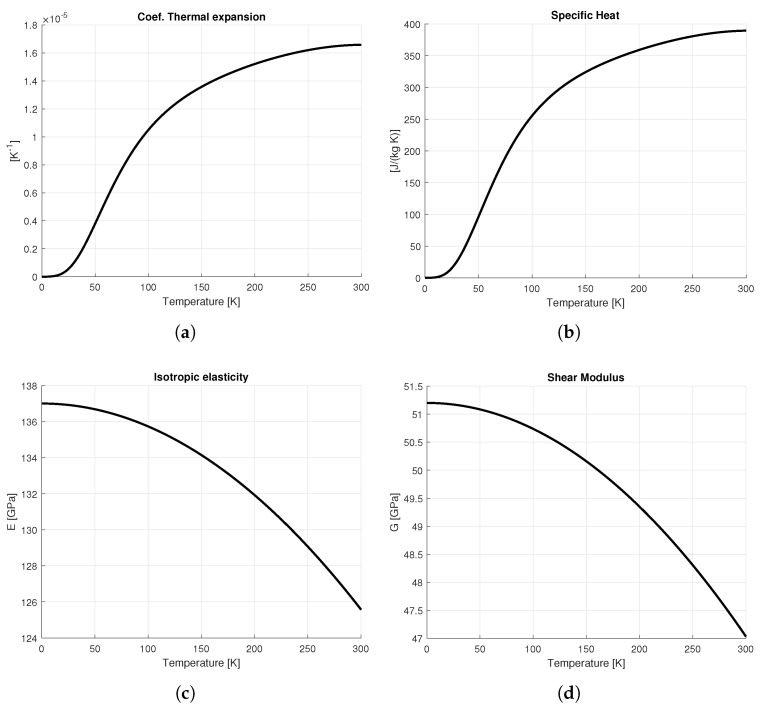
Material property graphs of OFHC copper (UNS C10100)—100RR as a function of temperature [10,12]. (**a**) Coefficient of Thermal expansion. (**b**) Specific heat. (**c**) Isotropic elasticity. (**d**) Shear Modulus.

**Figure A3:**
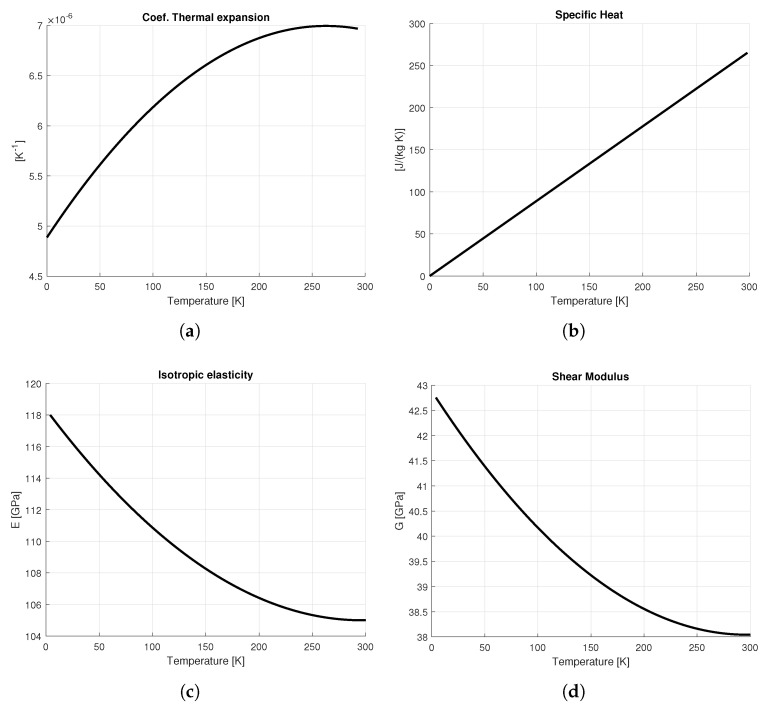
Material property graphs of Niobium-ES371110 as a function of temperature. (**a**) Coefficient of Thermal expansion. (**b**) Specific heat. (**c**) Isotropic elasticity. (**d**) Shear Modulus.

**Figure A4:**
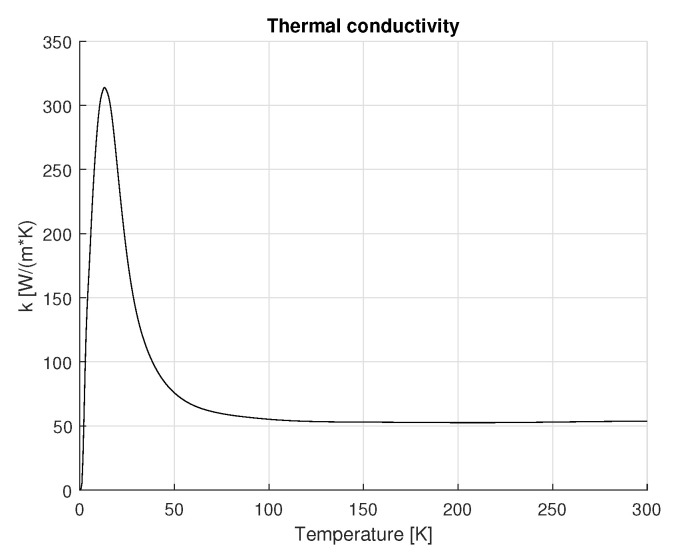
Graph of the thermal conductivity of Niobium-ES371110 as a function of temperature.

**Figure A5:**
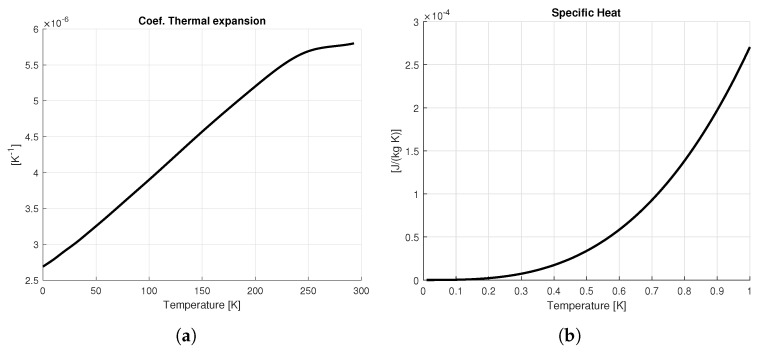
Material property graphs of lithium niobate as a function of temperature. (**a**) Coefficient of Thermal expansion. (**b**) Specific heat.

**Figure A6:**
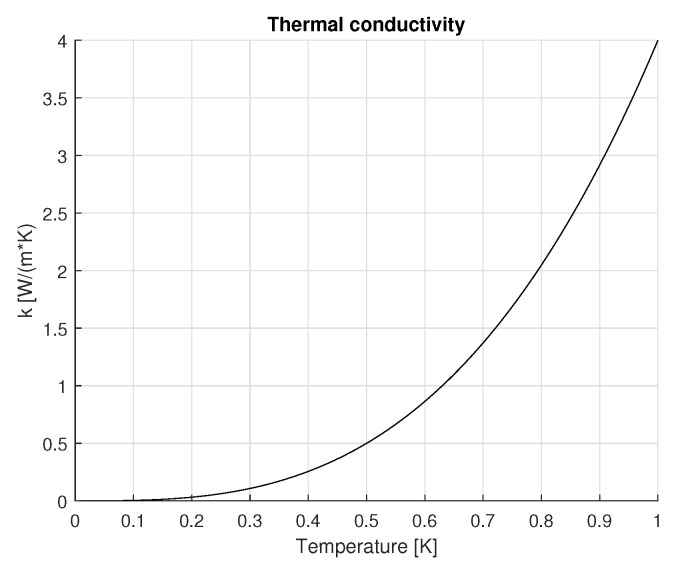
Graph of the thermal conductivity of lithium niobate as a function of temperature.

**Figure A7:**
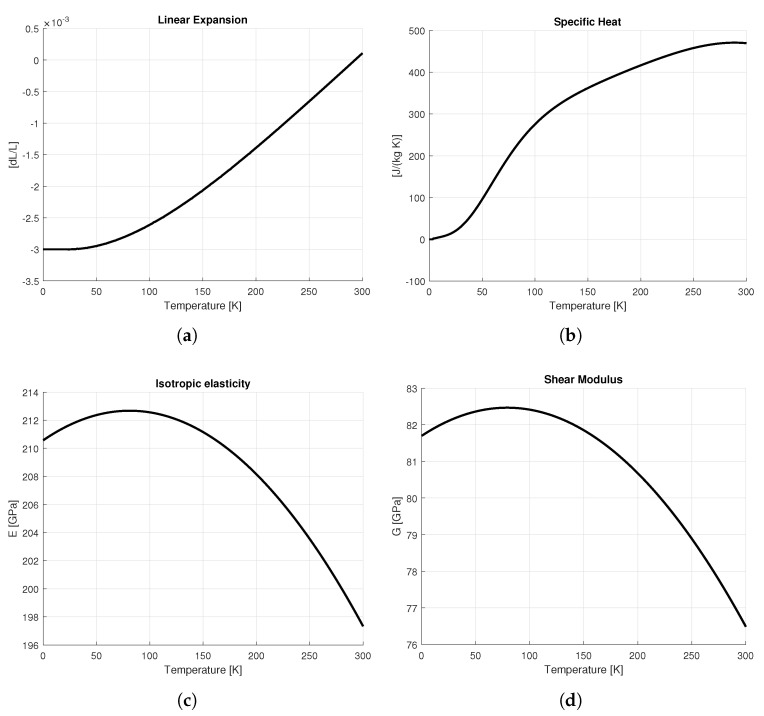
Material property graphs of 304 Stainless (UNS S30400) as a function of temperature [14,16,17]. (**a**) Linear expansion. (**b**) Specific heat. (**c**) Isotropic elasticity. (**d**) Shear Modulus.

**Figure A8:**
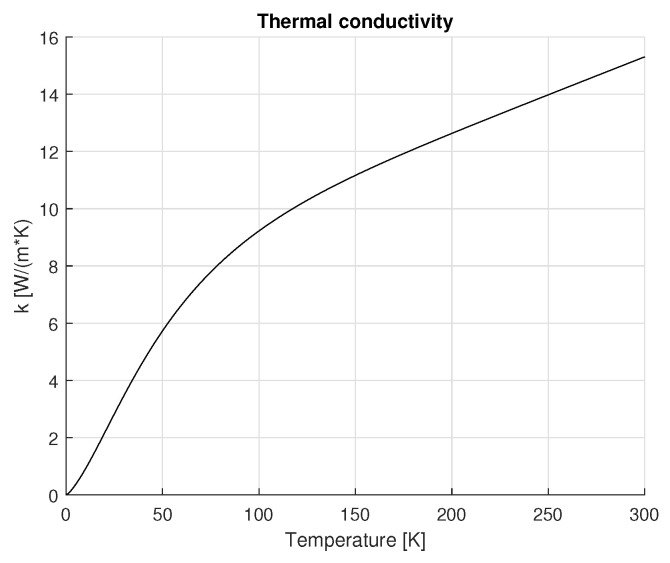
Graph of the thermal conductivity of 304 Stainless (UNS S30400) as a function of temperature [14,16,17].

**Figure A9:**
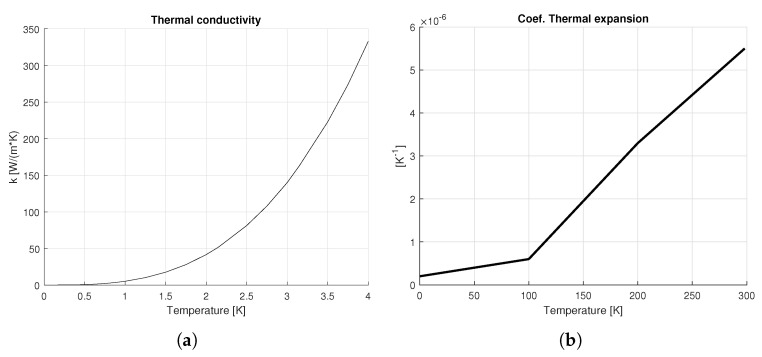
Material property graphs of Al_2_O_3_ as a function of temperature [12,19]. (**a**) Thermal conductivity. (**b**) Coefficient of Thermal expansion.

## Data Availability

All relevant data are included in the article. Additional data that support the findings of this study are available upon reasonable request from the authors.

## Appendix A. Materials

This appendix focuses on the material properties used in the simulations. For the temperature range between 4 K and 295 K, the material properties have been primarily derived from the resources provided by the Cryogenic Technologies Group at NIST (https://trc.nist.gov/cryogenics/index.html, accessed on 11 February 2025). For temperatures below 4 K, some data were available in the literature. In cases where no information on material properties below 1 K could be found, the values were extrapolated, as suggested in Ref. [7], to ensure consistency with the curve’s behavior near cryogenic temperatures.

### Appendix A.1. OFHC Copper (UNS C10100)-100RRR

OFHC Copper (UNS C10100)-100RRR is normally used in low-temperature structures when a combination of both physical (high conductivity) and mechanical (adequate strength) properties are needed. This type of copper has a density of 8960 kg/m^3^.

**Table A1:** Alloy Composition (Wt.%) [9].

Alloy No.	Designation	Description	Cu (% Min)	As	Sb	P	Te	Others
C10100	OFHC	Oxygen-free high thermal conductivity	99.99 (a)	0.0005	0.0004	0.0003	0.0010	(b)

(a) Copper is determined by the difference of impurity total from 100%. (b) Bi, max., 1 ppm (0.0001%); Cd, max., 1 ppm (O.X1%), Fe, max., 10 ppm (0.0010%); Pb, max., 5 ppm (0.0005%); Mn, max., 0.5 ppm (0.00005%); Hg, max., 1 ppm (0.00001%); Ni, max., 10 ppm (0.0010%); Oxygen, max., 5 ppm (0.0005%); Se, max., 3 ppm (0.0003%); Ag, max., 25 ppm (0.0025%); S, max., 15 ppm (0.0015%); Sn, max., 2 ppm (0.0002%); Zn, max., 1 ppm (0.0001%). Hg, max., 1 ppm (0.0001%); Zn, max., 1 ppm (0.0001%).

**Table A2:** Material property.

Density (kg/m^3^)	Tensile Yield Strength (MPa)	Tensile Ultimate Strength (MPa)
8960	180	310

Oxygen-free high thermal conductivity (OFHC) is a high-purity copper used in applications requiring high thermal and electrical properties. The theoretical residual resistivity ratio (RRR) could be calculated as:

$$R=\frac{\rho_{300K}}{\rho_{0K}},$$

where $\rho_{300K}$ is the resistivity of the material at room temperature and $\rho_{0K}$ is the resistivity of the material at temperatures close to absolute zero (0 K). The RRR is an essential measure of the purity and quality of copper, as impurities and defects can increase the resistivity of the material at low temperatures. In practice, the RRR value is determined through measurements at various temperatures, using models to estimate resistivity at 0 K. A high RRR value, such as 100, indicates a material of excellent quality, particularly suitable for applications in electronics, superconductors, and other fields where the conduction of heat and electricity is critical. Here are the graphs of the material properties used to obtain the results of this work.

### Appendix A.2. NIOBIUM 300RRR

The quantum transducer components are manufactured from high-purity niobium with a Residual Resistivity Ratio (RRR) of 300, a grade commonly adopted for superconducting applications such as those at CERN. The material conforms to ASTM B393-09e1 (R04220-Type 5) specifications in the fully annealed condition, with additional requirements for superconducting performance.

At cryogenic temperatures, the material properties are strongly temperature dependent. In particular, below 1 K the thermal conductivity exhibits a $T^{3}$ dependence.

**Table A3:** Limits for the main impurities (Max. Content Wt.%) [13].

Ta	W	Ti	Mo	H_2_	N_2_	O_2_	C	Metallic Impurities
0.050	0.007	0.005	0.005	0.0002	0.0010	0.0010	0.0010	0.003

**Table A4:** Material property.

Density (kg/m^3^)	Tensile Yield Strength (MPa)	Tensile Ultimate Strength (MPa)
8600	200	203

The following graphs illustrate the material properties utilized to obtain the results presented in this work.

### Appendix A.3. Lithium Niobate (LiNbO3)

Lithium niobate (LiNbO_3_) is a synthetic salt composed of niobium, lithium, and oxygen. Its single crystals are crucial in various applications, including optical waveguides, mobile phones, piezoelectric sensors, and optical modulators. This colorless solid is insoluble in water and has a trigonal crystal system. To enhance its resistance to optical damage, lithium niobate can be doped with magnesium oxide. Due to its advantageous optical, piezoelectric, electro-optic, elastic, photoelastic, and photorefractive properties, ferroelectric lithium niobate is extensively used in integrated and guided-wave optics. Known as LN crystal, lithium niobate boasts several benefits, such as chemical stability, non-hygroscopicity, high nonlinear optical, electro-optic and acoustic coefficients, availability in large sizes, and cost-effectiveness. Its high nonlinear optical coefficient makes it one of the best choices for low- to mid-power laser systems, while its strong electro-optic coefficient makes it a prevalent material in electro-optic modulation. The main limitation of lithium niobate is its low damage threshold, which can be mitigated by incorporating magnesium to form MgO crystals. LiNbO_3_ has no real yield stress (it does not plastically deform before fracture).

**Table A5:** Material property [14,15].

Density (kg/m^3^)	Isotropic Elasticity (GPa)	Shear Modulus (GPa)	Tensile Yield Strength (MPa)	Tensile Ultimate Strength (MPa)
4650	105	40.38	-	210

Below are the graphs of the material properties that were used to obtain the results of this work. The thermal conductivity and the specific heat capacity LiNbO_3_ exhibit a $T^{3}$ dependence at cryogenic temperatures.

### Appendix A.4. 304 Stainless (UNS S30400)

AISI 304 stainless steel is the benchmark for austenitic stainless steels, characterized by excellent corrosion resistance, outstanding ductility, and formability, as well as superior weldability. Its versatility in surface finishing—allowing for decorative and painted options—makes it suitable for a wide range of applications. This material can be used in environments ranging from cryogenic temperatures up to 900 °C, including the oil and chemical industries, metallurgical machinery, aerospace applications, food processing equipment, instruments, household appliances, and hardware production.

**Table A6:** Alloy Composition (Wt.%).

C (%Max)	Si (%Max)	Mn (%Max)	P (%Max)	S (%Max)	N (%Max)	Cr	Ni
0.07	0.75	2.00	0.045	0.030	0.10	17.5–19.5	8.0–10.5

**Table A7:** Material property.

Density (kg/m^3^)	Tensile Yield Strength (MPa)	Tensile Ultimate Strength (MPa)
7900	270	650

Below are the graphs of the material properties that were used to obtain the results of this work.

### Appendix A.5. Sapphire (Al2O3)

Synthetic Sapphire is a single crystal form of corundum, Al_2_O_3_, also known as alpha-alumina, alumina, and single crystal Al_2_O_3_. Sapphire is aluminium oxide in the purest form with no porosity or grain boundaries, making it theoretically dense. The combination of favourable chemical, electrical, mechanical, optical, surface, thermal, and durability properties make sapphire a preferred material for high performance system and component designs. For various semiconductor applications, sapphire is the best choice in comparison with other synthetic single-crystals.

**Table A8:** Material property at room temperature [18].

Density (kg/m^3^)	Young’s Modulus (GPa)	Shear Modulus (GPa)	Specific Heat Capacity (J/(g × K))	Ultimate Tensile Strength (MPa)
3960	370	151.64	763	400
